# Active bacterial core surveillance of the emerging infections program network.

**DOI:** 10.3201/eid0701.010114

**Published:** 2001

**Authors:** A Schuchat, T Hilger, E Zell, M M Farley, A Reingold, L Harrison, L Lefkowitz, R Danila, K Stefonek, N Barrett, D Morse, R Pinner, Active Bacterial Core Surveillance Team of the Emerging Infections Program Network

**Affiliations:** Centers for Disease Control and Prevention, Mailstop C23, 1600 Clifton Rd., Atlanta, GA 30333, USA. aschuchat@cdc.gov

## Abstract

Active Bacterial Core surveillance (ABCs) is a collaboration between the Centers for Disease Control and Prevention and several state health departments and universities participating in the Emerging Infections Program Network. ABCs conducts population-based active surveillance, collects isolates, and performs studies of invasive disease caused by Streptococcus pneumoniae, group A and group B Streptococcus, Neisseria meningitidis, and Haemophilus influenzae for a population of 17 to 30 million. These pathogens caused an estimated 97,000 invasive cases, resulting in 10,000 deaths in the United States in 1998. Incidence rates of these pathogens are described. During 1998, 25% of invasive pneumococcal infections in ABCs areas were not susceptible to penicillin, and 13.3% were not susceptible to three classes of antibiotics. In 1998, early-onset group B streptococcal disease had declined by 65% over the previous 6 years. More information on ABCs is available at www.cdc.gov/ncidod/dbmd/abcs. ABCs specimens will soon be available to researchers through an archive.


### Active Bacterial Core Surveillance of the Emerging Infections Program Network

**Anne Schuchat,* Tami Hilger,* Elizabeth Zell,* Monica M. Farley,† Arthur Reingold,‡ Lee Harrison,§ Lewis Lefkowitz,¶ Richard Danila,** Karen Stefonek,†† Nancy Barrett,‡‡ Dale Morse,§§ and Robert Pinner,* for the Active Bacterial Core Surveillance Team of the Emerging Infections Program Network**

*Centers for Disease Control and Prevention, Atlanta, Georgia, USA; †Georgia Emerging Infection Program (Georgia Department of Human Resources, Division of Public Health, Emory University School of Medicine, and the Atlanta Veterans Administration Medical Center) Atlanta, Georgia, USA; ‡California Department of Health Services and University of California Berkeley School of Public Health, Berkeley, California, USA; §Maryland Department of Health and Mental Hygiene and Johns Hopkins University School of Public Health, Baltimore, Maryland, USA; ¶Tennessee Department of Health and Vanderbilt University Medical Center, Nashville, Tennessee, USA; **Minnesota Department of Health, St. Paul, Minnesota, USA; ††Oregon Department of Human Resources, Portland, Oregon, USA; ‡‡ Connecticut Department of Public Health, Hartford, Connecticut, USA; and §§New York State Department of Health, Albany, New York, USA

Active Bacterial Core surveillance (ABCs) is a collaboration between the Centers for Disease Control and Prevention and several state health departments and universities participating in the Emerging Infections Program Network. ABCs conducts population-based active surveillance, collects isolates, and performs studies of invasive disease caused by *Streptococcus pneumoniae*, group A and group B *Streptococcus*, *Neisseria meningitidis*, and *Haemophilus influenzae* for a population of 17 to 30 million. These pathogens caused an estimated 97,000 invasive cases, resulting in 10,000 deaths in the United States in 1998. Incidence rates of these pathogens are described. During 1998, 25% of invasive pneumococcal infections in ABCs areas were not susceptible to penicillin, and 13.3% were not susceptible to three classes of antibiotics. In 1998, early-onset group B streptococcal disease had declined by 65% over the previous 6 years. More information on ABCs is available at www.cdc.gov/ncidod/dbmd/abcs. ABCs specimens will soon be available to researchers through an archive.

Bacterial infections are prototypical emerging diseases (1), and their recent history challenges the premature view that the battle against infectious diseases had been won. In the last 25 years, disease caused by multidrug-resistant *Streptococcus pneumoniae* became established on several continents, reaching the United States by the 1990s (2-4), and fatal infections caused by *S. pyogenes* (group A *Streptococcus*), a problem of the 19th century (5), have returned in toxic and necrotic forms (6). By the 1970s, group B *Streptococcus* replaced gram-negative bacteria and *Staphylococcus aureus* as the leading cause of sepsis in newborns (7,8). Researchers tackled the public health challenge of developing vaccines to protect children against the major causes of bacterial meningitis: *Haemophilus influenzae* type b, *S. pneumoniae*, and *Neisseria meningitidis* (9,10). A critical step for response to microbial adaptation is establishing a reliable tracking system. We describe active, population-based surveillance for serious bacterial infections that was established by the Centers for Disease Control and Prevention

Address for correspondence: Anne Schuchat, Centers for Disease Control and Prevention, Mailstop C23, 1600 Clifton Rd., Atlanta, GA 30333, USA; fax: 404-639-3970; e-mail: aschuchat@cdc.gov.

(CDC) as part of its response to emerging infectious diseases (11,12).

Active Bacterial Core surveillance (ABCs) was designed to estimate the burden of community-acquired invasive bacterial infections that typically manifest as sepsis and meningitis. The system determines incidence and trends of these diseases in a multistate population and uses molecular and microbiologic methods to characterize the organisms causing infection. As prevention strategies against some pathogens are used routinely (9,13,14), ABCs evaluates their impact and identifies missed opportunities for their application. Established in four states in 1995, ABCs now operates within the eight states of the Emerging Infections Program (EIP) network, representing a population of more than 30 million and ascertaining cases from more than 600 clinical microbiology laboratories. A ninth EIP state, Colorado, initiated ABCs during 2000. ABCs currently focuses on surveillance and special studies related to five pathogens: *S. pneumoniae*, *H. influenzae*, *N. meningitidis*, group A *Streptococcus* (*S. pyogenes),* and group B *Streptococcus* (*S. agalactiae).*

ABCs' predecessor was the active surveillance program for invasive bacterial diseases established in 1988 (also sponsored by CDC), which evaluated the efficacy of *H. influenzae* type b vaccines in infants (15), identified dietary risk factors for sporadic listeriosis (16,17), and compared the cost-effectiveness of strategies for preventing group B streptococcal disease in newborns (18). ABCs has expanded the scope of targeted conditions to address additional emerging infections such as necrotizing fasciitis (the so-called flesh-eating disease) and streptococcal toxic-shock syndrome, both severe manifestations of disease caused by group A *Streptococcus.* ABCs also now monitors the emergence of drug resistance in the community-acquired pathogen *S. pneumoniae*. ABCs is one of three core activities conducted by EIPs; the others are FoodNet (19) and the Unexplained Critical Illness and Death Project (20). This article, a progress report of the first 5 years of the EIP network's ABCs project, identifies easily accessible resources from this system for public health and infectious disease constituencies.

### ABCs Methods

In 1999, ABCs was conducted in Connecticut as well as in part or all of the following states: California, Georgia, Maryland, Minnesota, New York, Oregon, and Tennessee (Figure 1). (For certain pathogens, surveillance is conducted statewide in Georgia, Maryland, Minnesota, and Oregon). The total population under surveillance in 1998 ranged from approximately 17.4 million for *S. pneumoniae* to 30.4 million for *N. meningitidis*.

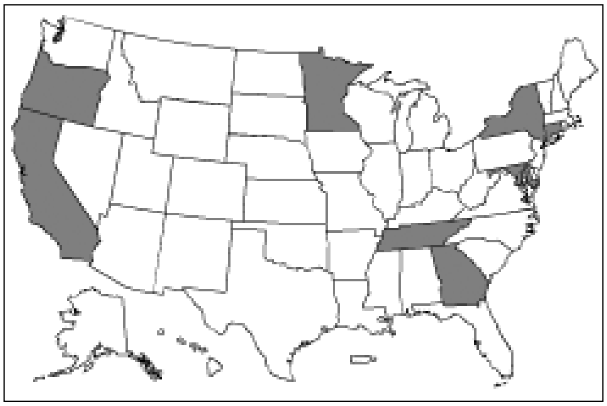

Figure 1. States included in Active Bacterial Core surveillance in 1999. Surveillance for all pathogens was conducted statewide in Connecticut but in selected counties only for some or all pathogens in the other states.

A case is defined as isolation of one of the five pathogens from a usually sterile site (e.g., blood, cerebrospinal fluid, pleural fluid) in a resident of one of the surveillance areas. Detailed methods of case-finding, data collection, and laboratory audits conducted within ABCs have been described (10,21). The key features are active ascertainment of cases by state-based surveillance officers, who make regular contact with microbiology or infection control practitioners in all clinical microbiology laboratories processing sterile site cultures for the surveillance area; collection of isolates of the specified pathogens for laboratory testing by ABCs personnel (Table 1); and semiannual audits of all participating laboratories to identify missed cases. Because the surveillance is population-based and cases identified by audits are included in the final database, ABCs data are used to monitor incidence of these diseases in a large, defined population. With the use of race- and age-adjustment, ABCs data also permit annual projections of the estimated incidence as well as the estimated number of cases and deaths occurring in the entire United States. For national projections, cases with unknown race

Table 1. Laboratory characterization of isolates collected as part of the Active Bacterial Core surveillance program

| Pathogen | Test(s) |
|---|---|
| Group A *Streptococcus* | *emm*- and T-typing for all invasive isolates; antimicrobial susceptibility testing of periodic samples |
| Group B *Streptococcus* | Serotyping and antimicrobial susceptibility testing of isolates for selected surveillance areas |
| *Haemophilus influenzae* | Serotyping of all isolates (a-f); molecular subtyping of isolates as part of special projects |
| *Neisseria meningitidis* | Molecular subtyping of isolates in conjunction with vaccine development; antimicrobial resistance on isolates periodically |
| *Streptococcus pneumoniae* | Antimicrobial susceptibility testing for all invasive isolates; serotyping on all invasive isolates since January 1, 1998; subtyping of a sample of isolates using genotyping methods |

are distributed by area, on the basis of reported race distribution for known cases within eight age categories. U.S. census data for counties under surveillance and natality data on live births are the sources of denominators for incidence calculations; the most recent year's population data available with age and race information at the county level are used for rate calculations.

Core surveillance activities include collecting epidemiologic and clinical data and characterizing isolates in terms of antimicrobial susceptibility, serotype or serogroup, and subtyping. ABCs also conducts special studies that use the surveillance infrastructure but require collection of additional data by chart review, patient interviews, or analysis of ABCs data together with complementary data sources. ABCs uses the following indicators to monitor performance: sensitivity of >90% for active surveillance (based on total cases detected by surveillance and the laboratory audit); collection of >85% of isolates from cases; and enrollment of 90% of eligible participants into special studies.

ABCs is overseen by a steering committee consisting of CDC and state EIP representatives as well as external advisors from the public health, infectious disease, and microbiology fields. These parties convey views from key constituents and annually evaluate the need to add or subtract pathogens for surveillance. In 1999, CDC's National Center for Infectious Diseases awarded $10.7 million through cooperative agreements to eight EIP states; approximately $2.5 million (23%) of these funds supported ABCs-related activities.

### Results

### Surveillance Highlights

In 1998, 6,992 cases of invasive disease caused by the five pathogens were reported from the eight sites. The rates of invasive disease (per 100,000) ranged from 1.0 for *N. meningitidis* to 24.1 for *S. pneumoniae* (Table 2). An estimated 97,000 invasive infections and 10,000 deaths per year in the United States are due to *S. pneumoniae*, group A and B streptococci,

Table 2. Incidence, case-fatality ratio, projected U.S. cases and deaths, and proportion nonsusceptible to penicillin of invasive disease identified in the Active Bacterial Core surveillance (ABCs), 1998

| | Group A *Streptococcus* | Group B *Streptococcus* | *Haemophilus influenzae* | *Neisseria meningitidis* | *Streptococcus pneumoniae* |
|---|---|---|---|---|---|
| Aggregate incidence[a] | 3.8 | 6.5 | 1.4 | 1.0 | 24.1 |
| Range by area[a] | 2.6 - 4.1 | 4.8 - 8.5 | 1.1 - 2.3 | 0.6 - 2.0 | 20.0-28.9 |
| Case- fatality ratio in ABCs areas | 12.2% | 9.5 % | 13.9% | 13.7% | 9.3% |
| Projected U.S. cases | 10,200 | 17,400 | 3,900 | 2,500 | 63,000 |
| Projected U.S. deaths | 1,300 | 1,700 | 500 | 400 | 6,100 |
| Penicillin nonsusceptibility[b] | 0 | 0 | - - | 1.1% | 25.0% |

[a]Incidence = cases per 100,000.
[b]Nonsusceptible includes isolates classified as either intermediate or resistant to penicillin. Results reflect testing of group A streptococcal isolates from 1997 (n=183) and group B streptococcal isolates from 1997 and 1998 combined (n=188).

*H. influenzae*, and *N. meningitidis*. Despite continued availability of effective antimicrobial agents for each pathogen, approximately 1 in 10 cases results in death (Table 2). Substantial geographic variation exists in the incidence of invasive infections caused by each pathogen (Table 2). Among invasive *S. pneumoniae* infections, the proportion caused by drug-resistant organisms was three times higher in some areas than others (4); 8.4% of invasive pneumococci from New York were fully resistant to penicillin (MIC $\geq$2.0), while 25.4% of isolates from Tennessee were penicillin resistant. No penicillin-nonsusceptible (intermediate or resistant) strains of group A or group B *Streptococcus* have been detected to date.

Recent temporal changes are most dramatic for invasive group B streptococcal disease among infants less than 1 week old (i.e., early-onset disease), which declined 65% from 1993 to 1998 (Figure 2), during a period when the incidence of disease in older infants and adults remained stable (22). Data from ABCs provide a reliable standard for evaluating alternative methods for surveillance of drug resistance in *S. pneumoniae*, including sentinel surveillance methods (4) and use of aggregate data from antibiograms from multiple hospitals (23). The recent emergence of serogroup Y meningococci, demonstrated by ABCs, suggests that vaccine companies should consider incorporating serogroup Y in new meningococcal vaccines. In addition, the diversity in the outer membrane proteins of serogroup B meningococcal strains suggests that vaccines against these proteins may not be efficient means of preventing endemic serogroup B meningococcal disease.

### Applied Research

The population-based collections of isolates from ABCs are used to evaluate subtyping methods (24), identify genetic mechanisms of antimicrobial resistance, determine vaccine formulations (25,26), and assess capsular switching among organisms (for vaccines based on capsular types) (27,28). ABCs has identified population-based risk factors for disease in various age groups (Table 3). A case-control study of invasive pneumococcal disease in young children showed that attendance at day care was associated with a substantial attributable risk for disease (29). A similar study of invasive pneumococcal disease in 18- to 64-year-old adults who were not immunocompromised identified active and passive smoking, in addition to household contact with a child in day care, as independent risk factors for disease (30). Models of age- and serogroup- or serotype-specific rates of invasive meningococcal and pneumococcal disease in the ABCs population have compared the potential impact of diverse immunization strategies for meningococcal and combined meningococcal-pneumococcal vaccines on disease prevention (32). The increased risk for pneumonia death occurring several days after illness onset associated with antimicrobial-resistant strains of *S. pneumoniae* was demonstrated by using multistate clinical and epidemiologic data from ABCs (33).

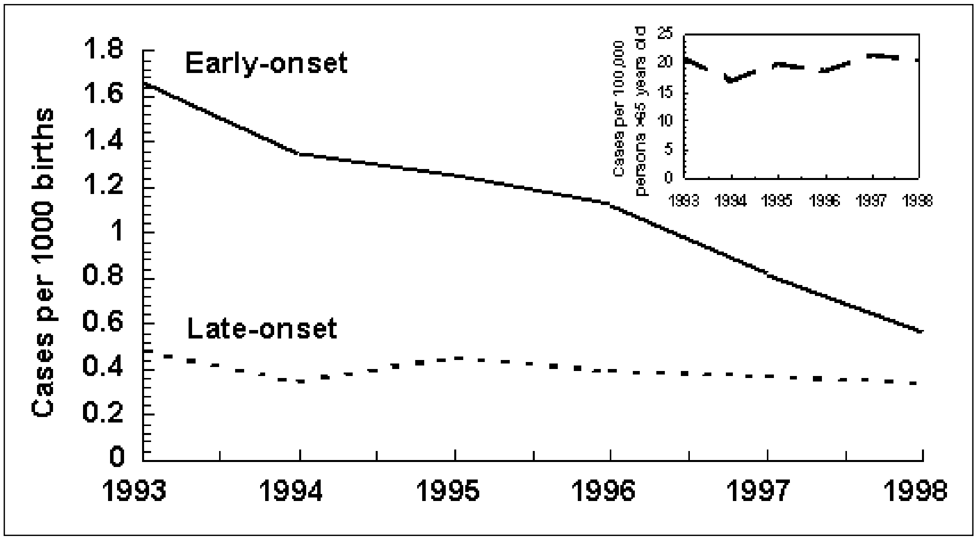

Figure 2. Invasive group B streptococcal disease in infants less than 1 week of age per 1,000 live births and in adults $\geq$65 years of age per 100,000 population, Active Bacterial Core surveillance, 1993-1998 (adapted from ref. 22).

Table 3. Population-based risk factors for invasive disease, identified by Active Bacterial Core surveillance

| Pathogen | Age group, other criteria | Factors associated with increased risk by multivariate analysis | References |
|---|---|---|---|
| *Streptococcus pneumoniae* | <5 years old | Child-care attendance, underlying conditions, lack of breast-feeding, household crowding | 29 |
| | 18-64 years old, not immuno-compromised | Active or passive smoke exposure, black race, chronic diseases, household contact with child in day care | 30 |
| *Neisseria meningitidis* | All ages | Active or passive smoke exposure, underlying conditions, steroid use, attendance at new school | 31 |
| Group B *Streptococcus* | <7 days old | Black race, low birth weight, maternal age <20 years | CDC, unpub. data |

### Infrastructure

ABCs provides participating state health departments active contact with all acute-care hospitals and reference microbiology laboratories in the surveillance area. This network provides an infrastructure for public health communication and education, as well as a network of key contacts available for response to new or emerging concerns. Periodic surveys of laboratories within ABCs determined the adequacy of methods used to detect group B *Streptococcus* from prenatal screening specimens (34), the computerization of clinical microbiology laboratories and readiness for electronic laboratory-based reporting, and the routine procedures used by ABCs laboratories to detect antimicrobial resistance among *S. pneumoniae*, *S. aureus,* and several other organisms (35). ABCs and other EIP personnel have provided assistance with multistate response efforts to determine the burden of Creutzfeldt-Jakob disease (36) and contributed to efforts to determine the rate of rotavirus vaccine-related intussusception (37). Further, the presence of ABCs personnel in state health departments and academic institutions has strengthened communication links required for accurate reporting and feedback.

### Prevention

Since publication of consensus guidelines for the prevention of group B streptococcal disease in newborns, ABCs assessed the implementation of prevention practices and identified opportunities for preventing more cases. ABCs showed that hospital obstetric programs' adoption of policies to prevent group B streptococcal infection increased significantly (38) and that hospitals that had adopted or revised a policy in 1996 had significantly fewer cases in 1997 (39). ABCs is also tracking the characteristics of newborn group B streptococcal cases that continue to occur despite prevention guidelines to determine whether these represent failures of intrapartum antibiotic prophylaxis or failure to offer such prophylaxis to mothers at risk. In several EIPs, pilot prevention programs are in place to identify efficient ways to reduce the incidence of disease caused by ABCs pathogens. These include a multifaceted program to reduce inappropriate antibiotic use in the Baltimore metropolitan area and efforts to promote pneumococcal polysaccharide vaccine in populations at high risk in Rochester, New York; Minneapolis-St. Paul, Minnesota; metropolitan Atlanta, Georgia; and Portland, Oregon. The Connecticut and Minnesota health departments conducted demonstration projects that integrated prevention of group B streptococcal disease into routine perinatal care, building on successes with hepatitis B perinatal prevention programs and contributing to reduction of perinatal HIV transmission (40,41).

### Discussion

Nearly 100,000 invasive infections and 10,000 deaths caused by ABCs pathogens occur annually in the United States. Because few states routinely collect data and isolates for all of these infections, ABCs helps monitor disease and evaluate prevention programs at the national level. ABCs has now developed robust estimates of the magnitude of disease and deaths attributable to the five invasive pathogens (Table 2). A number of future priorities have been identified that take advantage of the careful characterization of isolates associated with invasive infection. Licensure and introduction of a seven-valent conjugate vaccine against *S. pneumoniae* necessitate evaluation of the impact of this new prevention tool on target populations (Table 4). Of particular interest will be evaluating whether indirect effects similar to those seen with the Hib vaccine (42) occur. The large birth cohort under surveillance through ABCs and the longitudinal data on both early-onset cases and hospital policies for disease prevention offer the opportunity to compare the two alternative strategies for group B streptococcal prevention (screening-based vs. risk-based) through two studies during the next few years. ABCs will continue to contribute to tracking progress in Hib elimination, monitor for emergence of other serotypes of *H. influenzae*, and provide data on strain-specific disease (e.g., serotype, serogroup, outer membrane type). Such information is valuable for evaluating new vaccines for group B *Streptococcus* and serogroup B meningococcus. ABCs data will also be used to define clusters of invasive group A streptococcal disease and to model the impact of possible strategies and new formulations of pneumococcal vaccines targeted against pneumococcal pneumonia in adults (Figure 3) as well as vaccines targeted against invasive group A *Streptococcus* syndromes.

The molecular biology revolution and improved understanding of host-pathogen interactions offer great potential to advance knowledge about ABCs bacteria. Emerging antimicrobial resistance and other forms of pathogen adaptation (e.g., capsular switching) lend an urgency to such research. Specimens from invasive disease surveillance represent well-characterized, population-based collections with relevant clinical and demographic information. These provide a valuable resource for basic and applied research focused on issues as varied as new drug and vaccine development, evaluation of mechanisms of virulence and antimicrobial resistance, and genetic research. ABCs is planning to make these strains available to outside researchers and industry through a preserved collection. Such a specimen bank could provide a lasting legacy of the work of hundreds of infection control practitioners, clinical microbiology laboratories, and ABCs surveillance collaborators.

Table 4. Future priorities for Active Bacterial Core surveillance (ABCs) project

1. Define invasive group A *Streptococcus* clusters.
2. Determine effectiveness of screening vs. risk-based prevention strategies for perinatal group B streptococcal disease.
3. Determine feasibility of eliminating invasive disease caused by *Haemophilus influenzae* type b.
4. Quantify culture-negative, polymerase chain reaction-positive meningitis.
5. Measure direct and indirect effects of introducing a seven-valent pneumococcal conjugate vaccine.

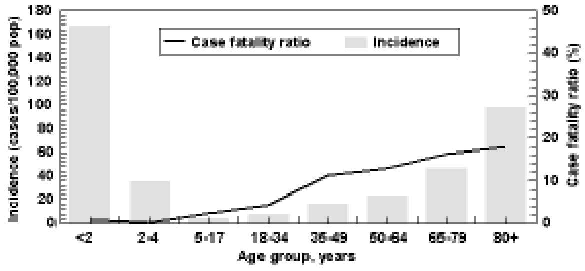

Figure 3. Age-specific incidence (per 100,000) and case-fatality ratio (percent) of invasive pneumococcal disease, Active Bacterial Core surveillance, 1998.

To ensure that ABCs' lessons learned within the EIP network reach other public health constituents, a number of efforts are under way. Additional details of the surveillance system and outreach materials are available at http://www.cdc.gov/ncidod/dbmd/abcs. Other educational materials are available at http://www.cdc.gov/ncidod/dbmd/gbs and at http://www.cdc.gov/ncidod/dbmd/antibioticresistance. For laboratories evaluating new strains of group A *Streptococcus*, genetic sequencing data of all strains described thus far are also available on the web. A similar site for meningococcal isolates is under development.

### Conclusions

ABCs is a model of collaboration between public health and academia. The system provides reliable data that can be used to address critical public health concerns, improve understanding of emerging infections, and help prevent the consequences of these infections. While the past 5 years have helped quantify the magnitude of disease caused by these pathogens and document increasing antibiotic resistance in some of them, the future provides several challenges. To remain a vital component in the nation's efforts to prevent and control emerging infectious diseases, ABCs will need to incorporate surveillance and research tools of the 21st century, including electronic laboratory-based reporting, genotyping of pathogens, and improved communication to promote behavioral change and adoption of practice guidelines.

### Acknowledgments

We thank the National Center for Infectious Diseases Emerging Infections Program and infection control practitioners and clinical microbiologists who collaborate on ABCs. We also thank the following additional members of the ABCs team: California: Duc Vugia, Gretchen Rothrock, Pam Daily, Lisa Gelling; Connecticut: James Hadler, Matt Cartter, Pat Mshar, Craig Morin, Aaron Roome, Heather Linardos; Georgia: Paul Blake, David Stephens, Kathryn Arnold, Wendy Baughman, Katherine McCombs, Sabrina Burden, Patricia Martell-Cleary, Mathew Sattah; Maryland: Margaret Pass, Diane Dwyer; Minnesota: Ruth Lynfield, Catherine Lexau, Jean Rainbow, Karen White, Lori Triden, Brenda Sayler; New York: Perry Smith, Shelly Zansky, Barbara Damaske, Nancy Bennett, Glenda Smith, Nellie Dumas, Brian Sauders, Hwa Gan Chang; Oregon: Paul Cieslak, Linda Duke; Tennessee: Allen Craig, William Schaffner, Brenda Barnes, Carolyn Gilmore; CDC: Katherine Robinson, Chris Van Beneden, Cynthia Whitney, Nancy Rosenstein, Carolyn Wright, Kathleen Shutt, Melissa Berkowitz, Falgunee Parekh, Richard Facklam, Bernard Beall, John Elliott, and Tanja Popovic.

Dr. Schuchat is chief of the Respiratory Diseases Branch at the Centers for Disease Control and Prevention. Her research interests include group B streptococcal disease, evaluation of vaccines against meningitis and pneumonia, and prevention of perinatal infections.

### References

1. Lederberg J, Shope RE, Oaks SCJ. Emerging infections: microbial threats to health in the United States. Washington: National Academy Press; 1992.
2. Centers for Disease Control and Prevention. Defining the public health impact of drug-resistant *Streptococcus pneumoniae*: report of a working group. MMWR Mor Mortal Wkly Rep 1996;45(No.RR-1):1-14.
3. Klugman KP. Pneumococcal resistance to antibiotics. Clin Microbiol Rev 1990;3:171-96.
4. Centers for Disease Control and Prevention. Geographic variation in penicillin resistance in *Streptococcus pneumoniae*—selected sites, United States, 1997. MMWR Mor Mortal Wkly Rep 1999;48:656-61.
5. Charles D, Larsen B. Streptococcal puerperal sepsis and obstetric infections: a historical perspective. Rev Infect Dis 1986;8:411-22.
6. Schwartz B, Facklam RR, Breiman RF. Changing epidemiology of group A streptococcal infection in the USA. Lancet 1990;336:1167-71.
7. Freedman RA, Ingram DL, Gross I, Ehrenkranz PA, Warshaw JB, Baltimore RS. A half century of neonatal sepsis at Yale, 1928 to 1978. Am J Dis Child 1981;135:140-4.
8. McCracken GH. Group B streptococci: the new challenge in neonatal infections. J Pediatr 1973;82:703-6.
9. Centers for Disease Control and Prevention. Recommendations for use of *Haemophilus influenzae* b conjugate vaccines and a combined diphtheria, tetanus, pertussis and *Haemophilus* b vaccine. Recommendations of the Advisory Committee on Immunization Practices (ACIP). MMWR Mor Mortal Wkly Rep 1993;42(RR-13):1-15.
10. Schuchat A, Robinson K, Wenger JD, Harrison LH, Farley M, Reingold AL, et al. Bacterial meningitis in the United States in 1995. N Engl J Med 1997;337:970-6.
11. Centers for Disease Control and Prevention. Addressing emerging infectious disease threats: a prevention strategy for the United States. Atlanta: Centers for Disease Control and Prevention; 1994.
12. Centers for Disease Control and Prevention. Preventing emerging infectious diseases: a strategy for the 21st century. Atlanta: Centers for Disease Control and Prevention; 1998.
13. Schuchat A, Whitney C, Zangwill K. Prevention of perinatal group B streptococcal disease: a public health perspective. MMWR Mor Mortal Wkly Rep 1996;45(No. RR-7):1-24.
14. Centers for Disease Control and Prevention. Prevention of pneumococcal disease: recommendations of the Advisory Committee on Immunization Practices (ACIP). MMWR Mor Mortal Wkly Rep 1997;46(RR-8):1-24.
15. Wenger JD, Pierce R, Deaver K, Plikaytis BD, Facklam RR, Broome CV. Efficacy of *Haemophilus influenzae* type b polysaccharide-diphtheria toxoid conjugate vaccine in US children aged 18-59 months. Lancet 1991;338:395-8.
16. Schuchat A, Deaver K, Wenger JD, Plikaytis BD, Mascola L, Pinner RW, et al. Role of foods in sporadic listeriosis. I: case-control study of dietary risk factors. JAMA 1992;267:2041-5.
17. Pinner RW, Schuchat A, Swaminathan B, Hayes PS, Deaver KA, Weaver RE, et al. Role of foods in sporadic listeriosis. II: microbiologic and epidemiologic investigation. JAMA 1992;267:2046-50.
18. Mohle-Boetani J, Schuchat A, Plikaytis BD, Smith D, Broome CV. Comparison of prevention strategies for neonatal group B streptococcal infection: a population-based economic analysis. JAMA 1993;270:1442-8.
19. Centers for Disease Control and Prevention. The Foodborne Diseases Active Surveillance Network, 1996. MMWR Mor Mortal Wkly Rep 1997;46:258-61.
20. Perkins BA, Flood JM, Danila R, Holman RC, Reingold AL, Klug LA, et al. Unexplained deaths due to possibly infectious causes in the United States: defining the problem and designing surveillance and laboratory approaches. Emerg Infect Dis 1996;2:47-53.
21. Zangwill KM, Schuchat A, Wenger JD. Group B streptococcal disease in the United States, 1990: report from a multistate active surveillance system. MMWR Mor Mortal Wkly Rep 1992;41(SS-6):25-32.
22. Schrag S, Zywicki S, Farley MM, Reingold AL, Harrison LH, Lefkowitz LB, et al. Group B streptococcal disease in the era of intrapartum antibiotic prophylaxis, 1993-1998. N Engl J Med 2000;342:15-20.

23. Chin AE, Hedberg K, Cieslak PR, Cassidy M, Stefonek KR, Fleming DW. Tracking drug-resistant *Streptococcus pneumoniae* in Oregon: an alternative surveillance method. Emerg Infect Dis 1999;5:688-93.
24. Beall B, Facklam R, Thompson T. Sequencing the *emm*-specific PCR products for routine and accurate typing of group A streptococci. J Clin Microbiol 1996;34:953-8.
25. Harrison LH, Dwyer DM, Johnson JA. Emergence of serotype V group B streptococcal infection among infants and adults. J Infect Dis 1995;171:513.
26. Blumberg HM, Stephens DS, Modansky M, Erwin M, Elliott J, Facklam R, et al. Invasive group B streptococcal disease: the emergence of serotype V. J Infect Dis 1996;173:365-73.
27. Swartley JS, Marfin AA, Edupuganti S, Liu LJ, Cieslak P, Perkins B, et al. Capsule switching of *Neisseria meningitidis*. Proc Natl Acad Sci USA 1997;94:271-6.
28. Gherardi G, Whitney C, Facklam R, Beall B. Major related sets of antibiotic-resistant pneumococci in the United States as determined by pulsed-field gel electrophoresis and *pbp1a-pbp2b-pbp2x*-dhf restriction profiles. J Infect Dis 2000;181:216-29.
29. Levine OS, Farley MM, Harrison LH, Lefkowitz L, McGeer A, Schwartz B. Risk factors for invasive pneumococcal disease in children: a population-based case-control study in North America. Pediatrics 1999;103:e28.
30. Nuorti JP, Butler JC, Farley MM, Harrison LH, McGeer A, Kolczak MS, et al. Cigarette smoking and invasive pneumococcal disease. N Engl J Med 2000;342:681-9.
31. Fischer M, Harrison L, Farley M, Lefkowitz L, McGeer A, Schuchat A, et al. Risk factors for sporadic meningococcal disease in North America. Philadelphia: Infectious Diseases Society of America; 2000.
32. Lingappa J, Zell E, Rosenstein N, Schuchat A, Perkins BA, Active Bacterial Core Surveillance Team. Impact of vaccination strategies using meningococcal conjugate vaccines in the United States. Philadelphia: Infectious Disease Society of America; 1999.
33. Feikin DR, Schuchat A, Kolczak M, Barrett NL, Harrison LH, Lefkowitz L, et al. Mortality from invasive pneumococcal pneumonia in the era of antibiotic resistance, 1995-1997. Am J Public Health 2000;90:223-9.
34. Centers for Disease Control and Prevention. Laboratory practices for prenatal group B streptococcal screening and reporting—Connecticut, Georgia, and Minnesota, 1997-1998. MMWR Mor Mortal Wkly Rep 1999;48:426-8.
35. Centers for Disease Control and Prevention. Laboratory capacity to detect antimicrobial resistance. MMWR Mor Mortal Wkly Rep 2000;48:1167-71.
36. Centers for Disease Control and Prevention. Surveillance for Creutzfeldt-Jakob disease—United States. MMWR Mor Mortal Wkly Rep 1996;45:665-8.
37. Centers for Disease Control and Prevention. Intussusception among recipients of rotavirus vaccine—United States, 1998-1999. MMWR Mor Mortal Wkly Rep 1999;48:577-81.
38. Centers for Disease Control and Prevention. Adoption of hospital policies for prevention of perinatal group B streptococcal disease—United States, 1997. MMWR Mor Mortal Weekly Rep 1998;47:665-70.
39. Factor SH, Whitney CG, Zywicki S, Schuchat A, the ABC Surveillance Team. Effects of hospital policies on the 1996 Group B streptococcal consensus guidelines. Obstet Gynecol 2000;95:377-82.
40. Roome A, Carley K, Melchreit R, Foye G, Hadler J. Testing pregnant women for HIV. A survey of obstetricians and review of patient prenatal/obstetric medical records—Connecticut 1996-1997. Conn Med 1999;63:541.
41. Lynfield R, Rubin M, White K, Schuchat A, Moore K, Osterholm M, et al. Prenatal HIV screening practices in Minnesota. Philadelphia: Infectious Diseases Society of America; 1999.
42. Adams WG, Deaver KA, Cochi SL, Plikaytis BD, Zell ER, Broome CV, et al. Decline of childhood *Haemophilus influenzae* type b disease in the Hib vaccine era. JAMA 1993;269:221-6.
